# Computed Tomography Enterography: Quantitative Evaluation on Crohn's Disease Activity

**DOI:** 10.1155/2018/7351936

**Published:** 2018-07-22

**Authors:** Jingyun Cheng, Hui Xie, Hao Yang, Ke Wang, Guobin Xu, Guangyao Wu

**Affiliations:** ^1^Department of Radiology, Zhongnan Hospital of Wuhan University, Wuhan, Hubei Province 430071, China; ^2^Department of Radiology, Zhongshan Hospital of Wuhan University, Wuhan, Hubei Province 430071, China; ^3^Department of Magnetic Resonance Imaging, Zhongnan Hospital of Wuhan University, Wuhan, Hubei Province 430071, China

## Abstract

**Objective:**

To explore the feasibility of computed tomography enterography (CTE) in the quantitative evaluation of the activity of Crohn's disease (CD).

**Methods:**

There were 49 CD patients with whole clinical, enteroscopy, and CTE data to be analyzed retrospectively. The patients were graded as inactive (0–2), mild (3–6), and moderate-severe group (>6) based on simplified endoscopic activity score for Crohn's disease (SES-CD). The differences in bowel wall thickening, mural hyperenhancement in the portal vein period, and the ΔCT values were analyzed among groups using ANOVA (analysis of variance) and *q* test. Then, the parameters were correlated with SES-CD, C-reactive protein (CRP), and erythrocyte sedimentation rate (ESR).

**Results:**

In the 49 patients, 13 ones were inactive, 19 ones were mild, and 17 ones were moderate-severe; the thickness of bowel wall, mural hyperenhancement in the portal vein period, and ΔCT value among groups were all significantly different (*P* < 0.001 in all). Correlative analysis showed that compared with the SES-CD, the bowel wall thickening (*r* = 0.564, *P* < 0.001), mural hyperenhancement in the portal vein period (*r* = 0.585, *P* < 0.001), and ΔCT value (*r* = 0.533, *P* < 0.001) were moderately correlated.

**Conclusion:**

The mural hyperenhancement in the portal vein period, bowel wall thickening, and ΔCT value can accurately and quantitatively assess the activity of CD lesions and are potential visual biomarkers of CD lesions.

## 1. Introduction

CD is an idiopathic, chronic, relapsing, transmural inflammatory disease that affects the entire gastrointestinal tract and has a tendency toward segmental distribution [1, 2]. Accuracy in grading of disease activity is crucial for treatment planning, prognosis assessment, and treatment efficacy. Clinical endoscopy is the gold standard for diagnosing and assessing CD activity [3]; nevertheless, its aggressive nature and risks associated with complications limit its long-term follow-up in the application of CD patients. CTE is widely used in clinical practice. It can reveal intestinal lesions, assess intestinal inflammation, and clearly show extraintestinal complications, such as internal fistula, abdominal abscess, mesenteric hypervascularity, cellulitis, and lymph nodes [4–6]. MPR (multiplanar reformation) and MIP (maximum intensity projection) can display the lesion and mesenteric vessels in multiple directions. CTE takes a short time and provides a good image quality, which is why it has become the most common choice for clinicians and patients. So far, few studies have made a quantitative assessment and grading lesion activity based on CTE imaging findings in CD patients; however, patients with different levels of activity have different treatment regimens. This study was designed to investigate the feasibility and application of CTE in quantitatively assessing and grading CD activity based on the simplified endoscopic activity score for CD (SES-CD).

## 2. Materials and Methods

### 2.1. Patients

Patients who underwent CTE in Zhongnan Hospital of Wuhan University from April 2016 to June 2017 were recruited to participate in the present study. Inclusion criteria were the following: (a) according to the consensus of diagnosis and treatment of inflammatory bowel disease [1]; CD diagnosis was established according to clinical, histologic, enteroscopy, and imaging evaluation; (b) intestinal dilatation showed good results, and it did not affect the assessment; (c) patients had to undergo enteroscopy within 2 weeks of CTE and have not received any new medication between the 2 examinations; and (d) enteroscopy and imaging data are complete. Exclusion criteria were the following: (a) the diagnosis of CD is not confirmed; (b) intestinal dilatation was poor and can impact assessment; (c) the interval between endoscopy and CTE examination exceeds two weeks, or the patients have received medical treatment during the time; and (d) the patients have contraindication to anisodamine. Clinical disease activity was assessed using the Harvey-Bradshaw index (HBI); C-reactive protein (CRP) and erythrocyte sedimentation rate (ESR) were examined between the 2 examinations.

### 2.2. Computed Tomography Enterography

According to the CTE protocol, patients were required to consume liquid or semiliquid diet the day before the procedure, to take polyethylene glycol electrolyte (PGE) solution for bowel cleaning the night before the procedure, and to take oral 1500 mL to 2000 mL of 2.5% mannitol solution (depending on patient's physique and comfort) 1 hour before the procedure. A 10 mg of anisodamine was slowly injected intramuscularly into the buttocks to induce gastrointestinal hypotonia 10 min before CTE. The patients with glaucoma, benign prostatic hyperplasia, and other contraindications were excluded from the study.

CTE was performed using Siemens 64-row dual-source spiral CT (Siemens Definition, Siemens Erlangen, Germany) from the top of the diaphragm to the lower edge of the pubic symphysis. The CT scan and contrast-enhanced scan were performed using a breath-hold technique. The arterial phase was scanned 20 sec to 30 sec after injection of the contrast agent, and the venous scan was performed 60 sec to 65 sec after injection of the contrast agent. Tube voltage was 120 kV, tube current was 160 mA~240 mA, pitch was 0.6 mm, and layer thickness was 5 mm. Enhanced scan was obtained using dual-tube high-pressure syringe injection of the nonionic contrast agent iohexol (350 mg I/ml, Yangtze River Pharmaceutical Company) in the amount of 1.5 ml/kg, with injection flow rate of 2.5 ml/s. After scanning, the CTE images were reconstructed with 1 mm thin layer, MPR, and MIP at the Siemens postprocessing station.

### 2.3. Enteroscopy

After routine cleaning of the intestine and intravenous anesthesia with propofol, enteroscopy (oral—into the jejunum about 350 cm away from the pylorus and the anal—into the ileum about 150 cm away from the ileocecal valve) was performed by experienced gastroenterologists. Two experienced gastroenterologists without knowledge of CTE results retrospectively analyzed endoscopic images and scored the most serious intestinal lesions according to SES-CD [3] including the following: the presence and size of ulcers, extent of ulcerated surface, extent of affected surface, and the presence and type of narrowings. The CD patients were graded as inactive (0–2), mild (3–6), or moderate-severe (≥7) by SES-CD. The results were used for data analysis.

### 2.4. CT Imaging Analysis

The CT images and reconstructed images were analyzed by two experienced abdomen radiologists (Xu and Wu) who were blinded to the clinical, laboratory, and endoscopic information. The recording was as follows: (a) location of lesion; (b) the thickness and enhanced patterns [7] of the most severe lesions of the bowel wall: A-type was multilayer (three or more), B-type was a double layer, formed by the significantly enhanced mucosa and low-density submucosa, C-type was a double layer but without mucosal enhancement, and D-type was evenly enhanced with no stratification; (c) the CT value of mural hyperenhancement in the portal vein phase (60~65 s) and ΔCT value (i.e., the CT value of mural hyperenhancement − nonenhanced CT value), ROI (region of interest) was placed in the thickened mucosa, avoiding submucosal edema and fluid in the intestine; (d) stenosis, peri-intestinal inflammation, mesenteric hypervascularity (comb sign), enlarged lymph nodes, abscess, fistula, and other lesions. The most severe lesion site of the bowel wall thickness, the CT values of mural hyperenhancement, and ΔCT value were taken as the average of the two measurements in the analysis, and while the results of the readings appeared controversial, the two radiologists conducted consensus reading and reached agreement.

### 2.5. Statistics

Statistical analysis was performed using the SPSS version 22.0 software (SPSS Inc., Chicago, IL). *P* < 0.05 was considered statistically significant. Qualitative data were expressed as percentage; quantitative data were tested with Kolmogorov-Smirnov test; normal distribution data were expressed as mean ± standard deviation; nonnormal distribution data were expressed with the median and quartile range (QR). The difference between parameters among groups was compared using ANOVA. The comparison of two means with samples was performed by *q* test (student Newman-Keuls test, SNK). The comparison of the qualitative data was performed with the chi-square test; the correlations of CTE parameters with SES-CD as the reference standard were analyzed using Pearson correlation. The intraclass correlation coefficient (ICC) was used to analyze the consistency between the two measurements of quantitative data.

## 3. Results

### 3.1. Study Population

A total of 49 patients (33 male, 16 female; mean age: 34.8 ± 13.0 year; disease duration: 1 month–23 year) were included according to inclusion criteria. Baseline characteristics of the patients are shown in Table 1.

### 3.2. CTE Findings

Among 49 patients, there were 25 cases of bowel obstruction or stenosis, among which 24 cases were the active group (including mild and moderate-severe groups), and the difference was statistically significant (*χ*^2^ = 13.3, *P* < 0.001). Furthermore, 7 cases of lymph node short diameter were beyond 10 mm, and all of them belonged to the active group. 32 cases showed mesenteric hypervascularity (comb sign), where 28 of them were active and 4 were inactive, and the difference was statistically significant (*χ*^2^ = 9.3, *P* = 0.005). 30 cases of intestinal lesions showed peri-intestinal inflammation, where 27 cases were active and 3 were inactive, and the difference was statistically significant (*χ*^2^ = 10.8, *P* = 0.002). The difference in bowel wall-enhanced patterns among inactive, mild, and moderate-severe groups was shown in Table 2. There was a significant difference between the inactive group and the mild group (*χ*^2^ = 15.7, *P* = 0.001), and the difference between the inactive and moderate-severe cases was statistically significant (*χ*^2^ = 14.1, *P* = 0.03). Nonetheless, there was no significant difference between the mild and moderate-severe cases (*χ*^2^ = 2.2, *P* = 0.536). In addition, 1 case of mesenteric abscess and 2 cases of intestinal fistula appeared as active.

### 3.3. Difference of CTE Parameters among Different Groups

**Figure 1:**
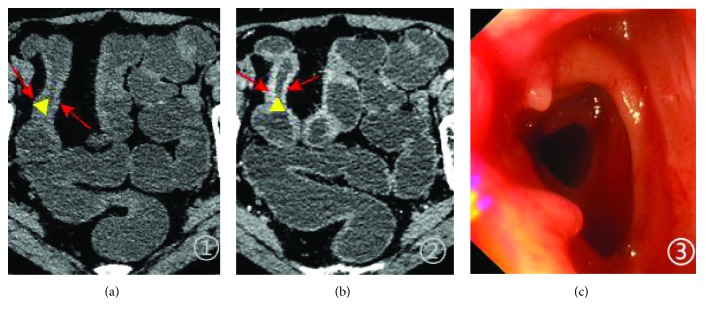
A 54-year-old woman with mild active CD, SES-CD of 4. CT plain scan shows the thickening of the bowel wall ((a) red arrowheads) and stenosis ((a) triangle) in the terminal ileum. Enhanced CT scan in the portal vein phase shows the thickened bowel wall from terminal ileum lesions which was evenly enhanced ((b) red arrowheads). The endoscopic image of the terminal ileum (c).

**Figure 2:**
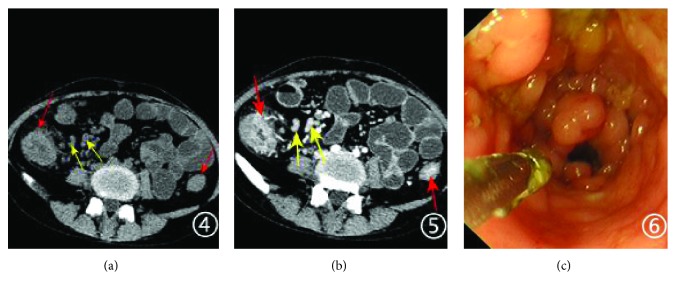
A 25-year-old women with mild active CD, SES-CD of 7. CT plain scan shows bowel wall thickening, peri-intestinal inflammation ((a) red arrowhead) in the ascending and descending colon, and mesenteric enlarged lymph nodes ((a) yellow arrowheads). Axial enhanced CT scan in the portal vein phase shows the thickened bowel wall of the ascending and descending colon and the stratified enhancement of thickened bowel wall ((b) red arrowheads) and mesenteric enlarged lymph nodes evenly enhanced ((b) yellow arrowheads). Endoscopic image is just like cobblestone (c).

According to the SES-CD, among 49 patients, 13 were inactive, 19 were mild Figures 1(a)–1(c), and 17 were moderate-severe Figures 2(a)–2(c). The thickness of the bowel wall, the CT value of mural hyperenhancement, ΔCT value, and the results of ANOVA among different groups were shown in Table 3. Further comparison between the two groups (*q* tests) shows that there are significant differences in the CT value of mural hyperenhancement between the groups. The thickness of the bowel wall was significantly different between the inactive and the mild (*P* = 0.002) and between the inactive and the moderate-severe (*P* < 0.001). There were significant differences in the ΔCT values between the inactive and the moderate-severe (*P* < 0.001) and between the mild and the moderate-severe cases (*P* = 0.001). The difference in thickness, the CT value of mural hyperenhancement, and ΔCT values between two groups is shown in Table 4.

### 3.4. Correlation between CTE Findings and Endoscopy and Laboratory

The bowel wall thickness (*r* = 0.564, *P* < 0.001), the CT value of mural hyperenhancement (*r* = 0.585, *P* < 0.001), and ΔCT value (*r* = 0.533, *P* < 0.001) were moderately correlated. The correlation coefficient between the thickness of the bowel wall and ESR and CRP was *r* = 0.542 (*P* < 0.001) and *r* = 0.452 (*P* = 0.002), respectively. Nonetheless, there was no correlation between the CT value of mural hyperenhancement and ΔCT value and ESR and CRP. In addition, the correlation coefficient between HBI and SES-CD was *r* = 0.590 (*P* < 0.001).

### 3.5. Interobserver Agreement

The intraclass correlation coefficient was 0.83 (*P* < 0.001) for bowel wall thickness, 0.83 (*P* < 0.001) for the nonenhanced CT value, and 0.90 (*P* < 0.001) for the CT value of mural hyperenhancement.

## 4. Discussion

Now, CTE has been widely used in the clinic, because of the following advantages: the short examination time, convenient procedure for the patients, good imaging quality, and multidirectional display of CD lesions and mesenteric vascular conditions.

Our study showed that bowel wall thickening, mural hyperenhancement, and the ΔCT value were moderately correlated with SES-CD, which was in line with the results of some studies about CTE [4, 8] and magnetic resonance enterography (MRE) [9, 10]. Pathologically, larger ulcers and a wider area of involvement in the CD often indicate a more pronounced presence of tissue hyperplasia and inflammatory cell infiltration [1, 2], which are manifested radiologically as bowel wall thickening and prominent mural hyperenhancement. A study by Wu et al. [4] revealed that after effective treatment, bowel wall thickening and mural hyperenhancement would be significantly decreased. Wu et al. [4] also found that bowel wall thickening and mural hyperenhancement were significantly correlated with CD lesion activity. However, the above studies did not grade the activity of the lesion nor did it compare the differences in mural hyperenhancement, bowel wall thickening, and ΔCT values between different activity groups. Our data showed that mural hyperenhancement was significantly different between groups; there was a significant difference in bowel wall thickening between the nonactive and the active but no significant difference between the mild and the moderate-severe. Possible explanation is that disease duration, recurrence, and other factors may affect bowel wall thickening. There were significantly different ΔCT values between the moderate-severe and the mild or the nonactive but no significant difference between the nonactive and the mild. Perhaps due to mild inflammatory response, the CT values of mild lesions were slightly elevated in the CT plain scan, when in the portal vein period, and the CT values of mural hyperenhancement are also not significantly increased. Our study also showed that bowel wall thickening was moderately correlated with ESR and CRP, generally considering that they are used to reflect the degree of inflammation in the clinic. The results suggested that bowel wall thickening might indirectly reflects the activity of the lesion. However, CRP and ESR levels are also associated with age, gender, and body mass index, with 20% of healthy subjects not having elevated CRP under inflammatory conditions [11], possibly explaining that there was no significant correlation between mural hyperenhancement and ΔCT values and ESR and CRP.

In our study, we found that stenosis was 25 cases, of which 24 cases were active, mesenteric hypervascularity (comb sign) was 32 cases, of which 28 cases were active, and 8 cases showed mesenteric enlarged lymph nodes, which were active. Several studies have reported that stenosis was related to the activity of the lesion [4, 12]. Chaudhry et al. [12] found that stenosis and the other complications, such as fistula and abscess, were reciprocal causation and affected the prognosis. Sakurai et al. [13] reported that comb sign and mesenteric enlarged lymph nodes were important predictors of mucosal ulceration; It is worth noting that the size of the ulcer and affected area is the most important part of the CD endoscopy score. Several studies [13–16] reported that comb sign not only reflected the degree of the lesion activity but also had a certain specificity in the CD diagnosis. As a result, mesenteric enlarged lymph nodes and comb sign can reflect the activity of the lesion. There were 30 cases showing peri-intestinal inflammation, of which 27 were active. When the lesion is in the active, peri-intestinal increased inflammatory exudates made peri-intestinal inflammation obvious. Choi et al. [7] divided enhanced patterns of wall thickening into four types. Our data showed that C-type and D-type were mainly in the nonactive, whereas A-type and B-type were in the active. These results are consistent with the results reported in some studies [4, 14]. During the active phase, the mucosal layer of the lesion is enhanced significantly as a result of the inflammatory reaction. The muscularis and serosal layers are moderately enhanced; however, the submucosa is usually not enhanced obviously due to edema. Nonetheless, the stratification is not a characteristic of CD and other intestinal inflammatory lesions such as intestinal tuberculosis and ulcerative colitis; they also have a similar performance [14]. CD is a chronic penetrating disease. Cellulitis, abscess, and fistula are more likely to occur compared with other inflammatory diseases. When the abscess, cellulitis, or fistula are found in the mesenteric, there is a high possibility for diagnosing CD. In this study, 1 case of mesenteric abscess and 2 cases of intestinal fistula revealed active CD.

However, this study has some limitations. Firstly, this is a retrospective study, where the bowel of study populations was not completely segmented and the sample size was still small. Secondly, although CTE is comparable to MRE in the diagnosis and activity assessment of CD [5, 8], still it is deficient in the diagnosis of fibrotic lesions and difficult to identify fibrosis or inflammation, which component predominates in the thickening or stenosis of the intestinal wall. In contrast, recent studies [17, 18] have reported that MR enhancement and special sequence can further identify the causes of bowel wall thickening. Although iterative reconstruction was used to lower the CT dose during the CT scan, there was no specific statistical analysis of the CT dose. Finally, although quantitative studies of bowel wall thickening and mural hyperenhancement have been performed in this study, quantitative CTE scoring systems like the magnetic resonance index of activity (MaRIA) [19] and Clermont's score [20] have not yet been developed and further multicenter studies of large samples are needed.

## 5. Conclusion

In conclusion, the mural hyperenhancement in the portal vein period, bowel wall thickening, and ΔCT value can accurately and quantitatively assess the activity of CD lesions and are potential visual biomarkers of CD lesions.

## Figures and Tables

**Table 1:** Characteristics of the Crohn's disease patients included in the study.

	*N* = 49
Gender (male/female)	33/16
Age, *x* ± *S*, year	34.8 ± 13.0
Disease duration: *M* (QR), month	16.8 (6.5, 45.9)
Interval, day	
≦7	42
7~14	7
Disease location	
Terminal ileum	21
Colorectal	4
Ileal and colon	16
Wide small intestine	8
HBI, *x* ± *S*	7.8 ± 3.3
CRP, *M* (IQR), mg/L	27.7 (6.5, 45.9)
ESR, *x* ± *S*, mg/L	30.6 ± 26.5
SES-CD	5.1 ± 2.6 (0, 12)

**Table 2:** Enhanced patterns among inactive, mild, and moderate-severe.

	Inactive	Mild	Moderate-severe
A	0	6	8
B	0	4	4
C	3	6	2
D	10	3	3
Total	13	19	17

**Table 3:** The results of ANOVA about bowel wall thickening, CT plain scan value, mural hyperenhancement, and ΔCT value among different groups.

	Thickness (mm)	CT plain scan (HU)	Mural hyperenhancement (HU)	ΔCT value (HU)
Nonactive (13)	5.9 ± 1.6	38.4 ± 4.6	72.4 ± 8.7	34.0 ± 8.8
Mild (19)	8.0 ± 2.3	41.6 ± 3.9	80.5 ± 7.4	38.9 ± 7.0
Moderate-severe (17)	8.9 ± 1.4	40.5 ± 3.2	89.2 ± 8.5	48.7 ± 8.9
*F* (*P* value)	10.5 (<0.001)	2.7 (0.079)	14.8 (<0.001)	12.8 (<0.001)

**Table 4:** Comparison in thickness, mural hyperenhancement, and ΔCT values between the two groups (significance).

	Thickness	Mural hyperenhancement	ΔCT value
Inactive versus mild	0.002	0.010	0.104
Inactive versus Moderate-severe	<0.001	<0.001	<0.001
Mild versus moderate-severe	0.156	0.003	0.001

## Data Availability

The data that support the findings of this study are available on request from the corresponding author (Guangyao Wu). The data are not publicly available because the aforementioned data containing information that could compromise research participant privacy.

## Abbreviations

CTE: Computed tomography enterography
SES-CD: Simplified endoscopic activity score for CD
CD: Crohn's disease
HBI: The Harvey-Bradshaw index
CRP: C-reactive protein
ESR: Erythrocyte sedimentation rate
MPR: Multiplanar reformation
MIP: Maximum intensity projection
PGE: Polyethylene glycol electrolyte.
