# Corrigendum to “Computed Tomography Enterography: Quantitative Evaluation on Crohn's Disease Activity”

**DOI:** 10.1155/2018/9286014

**Published:** 2018-11-05

**Authors:** Jingyun Cheng, Hui Xie, Hao Yang, Ke Wang, Guobin Xu, Guangyao Wu

**Affiliations:** ^1^Department of Radiology, Zhongnan Hospital of Wuhan University, Wuhan, Hubei Province 430071, China; ^2^Department of Magnetic Resonance Imaging, Zhongnan Hospital of Wuhan University, Wuhan, Hubei Province 430071, China

In the article titled “Computed Tomography Enterography: Quantitative Evaluation on Crohn's Disease Activity” [1], the affiliation of the fifth author is incorrect as it should be the same as the first affiliation. The corrected affiliations are shown above.

## References

[1] Cheng J., Xie H., Yang H., Wang K., Xu G., Wu G. (2018). Computed tomography enterography: quantitative evaluation on Crohn’s disease activity. *Gastroenterology Research and Practice*.

